# Mad or bad: Psychiatry’s foundational divide and the illusion of binary thinking

**DOI:** 10.1192/j.eurpsy.2025.10054

**Published:** 2025-06-30

**Authors:** Giovanni Stanghellini

**Affiliations:** 1 Department of Health Sciences, University of Florence, Florence, Italy; 2 Centro de Estudios de Fenomenologia y Psiquiatria, Universidad “Diego Portales”, Santiago, Chile

**Keywords:** dialectical model, mad-bad divide, health, mental health ethics, Philippe Pinel, public perception

## Abstract

This paper revisits Philippe Pinel’s (1745–1826) psychiatric legacy, on the occasion of the 200th anniversary of his death, to challenge the enduring dichotomy between madness and criminality. While Pinel is celebrated for separating the insane from the criminal, his deeper insight – that madness is always partial and never fully negates agency – has been largely overlooked. Drawing on this dialectical view, the paper critiques the persistence of rigid classifications in psychiatry and forensic contexts. It argues for a model of mental illness as a dynamic interplay between vulnerability and self-awareness, with profound implications for clinical practice, legal judgment, and public perception. By highlighting psychiatry’s double bind – caught between therapeutic nuance and legal absolutism – the paper calls for a renewed ethical stance that embraces complexity and reclaims psychiatry’s role as a bridge-builder rather than a boundary enforcer.

## Introduction

The year 2026 marks the 200th anniversary of the death of Philippe Pinel (1745–1826), believed to be one of the founding fathers of modern psychiatry. Modern psychiatry was born, in part, from a crucial distinction: madness is not a crime. Pinel’s late 18th-century reform did more than remove chains from the mentally ill – he separated the insane from the criminal, shifting their treatment from punishment to care [1]. Yet the binary logic this distinction introduced – mad or bad, ill or responsible – still haunts contemporary psychiatric and legal practice. This paper revisits Pinel’s often-overlooked insight that madness is never total and that even in psychosis, a person’s capacity for self-awareness and relation at least partially remains. Building on this dialectical view, the paper critiques the enduring dominance of rigid classifications in psychiatry and argues for a more nuanced model – one that sees mental illness not as a fixed state, but as a dynamic interplay between vulnerability and agency. Such a shift has wide-ranging implications: for how we diagnose and treat, how we legislate mental capacity, and how society understands the boundaries between sanity, suffering, and responsibility.

## Mad or bad: The foundational divide of modern psychiatry

It is commonly acknowledged that the birth of modern psychiatry coincides with the beginning of the 19th century and the theoretical and clinical legacy of Philippe Pinel [2] – the physician who famously “freed the mad from their chains.” This is the hagiographic version, but not for that reason inauthentic.

Pinel’s gesture not only restored human dignity to people suffering from madness but also clearly distinguished them from criminals. To the former he reserved medical treatment, leaving to the latter the attention of the justice system and its functions of mere surveillance and punishment. The human dignity of the mad person, and the treatment they are to receive – that is, *care* – is clearly separated from what is supposed to be due to the criminal: isolation, aimed at preventing further harm. The “mad or bad” dichotomy – still echoed in legal contexts – was historically used to draw a line between those deserving of care and those subject to punishment. It served to rescue the insane from the carceral universe of the prison and to create spaces and forms of care tailored to the specific illness that afflicts them. Yet, it risks oversimplifying the complex interplay between psychopathology and responsibility.

Around the same historical period – namely, the Enlightenment, characterized by the values of freedom, equality, and fraternity – the idea also emerged that criminals, too, should have their human dignity restored – along with the right to be reintegrated into civil society, and the corresponding duty of society to reintegrate them. Imprisonment would thus serve not only to surveil and punish but also to rehabilitate [3].

The restoration of human dignity to the insane therefore goes hand in hand with their distinction from the criminal. From this fundamentally dichotomous premise – either/or, mad or bad – a body of jurisprudence developed that recognized in the mad person, unlike the criminal, someone incapable of understanding and willing. On one side, then, madness; on the other, crime. On one side, evil in a psychopathological sense, i.e., illness that deprives the mad of the capacity for intent and will; on the other, evil in a moral sense, i.e., wickedness.

## “Partial madness” as a foundational principle

I would like to draw attention, however, to the hypothesis that this dichotomous approach is, in the thought of the father of modern psychiatry, only apparent. Pinel affirms, argues, and documents that madness is always *partial* [4]. No mad person is entirely mad, nor remains the same over time. This notion of the “partiality of madness” might seem secondary to the primary distinction between the criminal and the mad, but it is precisely on this premise that psychiatric institutions are founded – not as mechanisms of surveillance, but of care.

Pinel’s idea that madness is always partial opens a conceptual space for care: the patient remains a person, at least in part capable of agency, despite their vulnerability. The principle of “partial madness” highlights the coexistence of vulnerability and agency within the individual. Central to Pinel’s insight is the idea that mental illness never wholly consumes the individual. Even in severe mental health conditions, aspects of self-awareness, reasoning, and agency persist, allowing the person to engage with their experience and surroundings. This coexistence of vulnerability and capacity forms the foundation of a dialectical view of mental illness, opening space for care and dialogue rather than mere categorization [5].

This principle underpins modern psychotherapeutic and community psychiatry – as well as the United Nations *Convention on the Rights of Persons with Disabilities* [6]. The person with mental disorders remains a person precisely because of the persistence of a part of the self capable of taking a stance in relation to their psychopathological wound. Total madness would consist of the complete abolition of this “healthy” part – capable, that is, of understanding and willing – in relation to the “ill” or vulnerable part [7].

Thus, alongside a dichotomous conception that distinguishes criminal from mad, there is a dialectical conception of mental illness: what we call mental illness is the variable result of a dialectic between the person vulnerable to madness and their vulnerability. Psychopathological forms, courses, and outcomes vary depending on the relationship between the person – their self-awareness, stance, resilience, and ability to care for themselves – and their vulnerability to madness. Ultimately, this hinges on their (however partial) ability to understand and will.

To be fair, the dichotomous tendency still thrives in certain areas of contemporary psychiatry, as evidenced by various diagnostic manuals. The persistence of dichotomous thinking is especially evident in sectors of psychiatry that seek rigid boundaries between different psychopathological syndromes and equally clear prognoses regarding their development and outcomes.

## The shift from dichotomy to dialectics

Despite the prevalence of this dichotomous model in some areas of modern psychiatry, the dialectical conception of madness has had major consequences in the more recent history of the field – especially in its community-based and psychotherapeutic branches – and, more broadly, in how we think about the human condition and its relationship with madness [8].

Let us start with this latter point: at the turn of the 19th and 20th centuries, it became apparent that the human condition cannot be conceived or defined without accounting for its vulnerability to madness. This has been the core message of more than a century of psychopathological studies, philosophical reflections, and artistic and literary creations (not to mention so-called minor arts such as cinema or pop music) [9]. Few, if any of these representations of humanity, centered on its vulnerability to madness, adopt a dichotomous perspective. Rather, they more or less explicitly affirm a dialectical view in which the boundaries between health and illness and “evil” in the moral and psychopathological sense are fluid and sometimes indistinct.

This intellectual tradition does not provide answers about where to draw the line between mental health and illness. On the contrary, it raises questions – mostly unanswerable ones – that confront us with the mystery of the human condition, suspended between freedom and unfreedom. That seems to be the hallmark of the human condition – and certainly the hallmark of how a significant part of contemporary culture represents it [10].

Even on the clinical level, Pinel’s dialectical framework has had profound implications. In this context, “dialectics” can be defined as a method of understanding phenomena as dynamic and interrelated processes, where opposing forces or conditions – such as vulnerability and agency in mental illness – interact and shape each other, rather than existing as fixed, separate categories. This contrasts with the traditional binary approach that views madness and morality as mutually exclusive states.

Community psychiatry models would be unthinkable without the theoretical foundations provided by the principle of “partial madness” and a dialectical framework. The core theoretical assumption can be summed up as follows: care is a dialogue between the caregiver and the person experiencing madness – aimed at establishing or reestablishing a dialectic between the person and their vulnerability to madness [11]. Consider two patients with similar psychotic symptoms: one finds support in a therapeutic setting and retains a sense of self-reflection; the other, isolated and untreated, deteriorates. It is not just the illness, but the relationship with the illness that shapes the outcome. It is not just the illness itself, but the quality of the relationship between the person and their illness – and between patient and caregiver – that shapes the outcome. This perspective invites a rethinking of what we mean by “recovery”: not the mere elimination of symptoms or the pathogenic cause, but the attainment of a new, more resilient equilibrium within a person’s inherent vulnerability. This is achieved by moderating the intensity of distressing experiences and reducing their pervasiveness, thereby allowing for a deeper exploration of their existential significance.

While this outlook is undeniably optimistic, its strength lies in promoting a more balanced therapeutic stance. Viewing patients as passive sufferers of illness risks fostering asymmetric relationships, narrowing our capacity to engage with their point of view, limiting our grasp of their lived reality, and confining us within the restrictive lens of professional detachment. In contrast, a dynamic approach enables a more reciprocal therapeutic alliance – one focused on supporting the patient’s own efforts toward healing, grounded in the process of self-understanding.

## Legal demands, clinical realities: Psychiatry’s dilemma beyond dichotomy

Throughout this paper, I have argued for a dialectical understanding of mental illness that resists simple binary classifications. From the dialectical perspective, all dichotomous views – here health, there illness, here mad, there bad – are ruled out. Yet, in forensic psychiatry, these binaries remain deeply entrenched. Now, here is my reasoning: if, in forensic settings, psychiatrists are asked to determine whether a person is healthy or ill, is it possible to formulate this question in non-dichotomous terms? Can a psychiatrist ethically answer a legal question that demands a binary where clinical experience sees a spectrum? This is not just a technical dilemma – it is a question about the very image of psychiatry in the public sphere.

This question leads immediately to a second one: what are the consequences, for the public image of psychiatry, of posing this question in dichotomous terms? At the very least, it will generate confusion in the public mind: on the one hand, psychiatrists claim that mental illness is dialectical in nature, and therefore treatable; on the other, they conform to a legal institution that affirms a dichotomous division between health and illness. A dichotomous stance may also contribute to reinforce the idea that the task of psychiatry is to protect society from the danger that madness may pose, rather than to protect persons suffering from mental conditions from the exclusion and stigma sometimes imposed by society.

Viewed in these terms, the figure of the psychiatrist appears at least ambiguous. And their public image, at best, disorienting – if not outright stigmatized. The stigma may stem from the perception of psychiatrists as those who wield the power, in legal contexts, to draw a sharp line between health and mental illness – while at the same time, in therapeutic contexts, preaching that the boundaries are fluid and dependent on the relationship between the person and their vulnerability, and between the vulnerable person and the care institutions, as well as their social environment.

## Conclusion: Psychiatry’s double-bind dichotomies, discredit, and the demand for bridges

In forensic settings, psychiatry is often called upon to deliver clear-cut judgments – healthy or ill – despite the inherently complex and spectrum-based nature of mental health. This tension between legal demands for binary answers and clinical realities that resist such neat categorization poses a fundamental dilemma. Beyond a technical challenge, it raises profound questions about the ethical responsibilities of psychiatrists and the public’s perception of their role. How can psychiatrists navigate this dilemma and what are the implications for its legitimacy, stigma, and future direction?

I merely wished to highlight this potential contradiction, without claiming to resolve it. It is a painful contradiction and perhaps lies at the root of psychiatry’s potential or actual discredit. The stigma to which our discipline is still subject may have deep roots in this ambiguity.

Is psychiatry’s role to dig trenches or to build bridges? Psychiatry must choose: does it build bridges between health and illness, or does it dig trenches that trap both patients and practitioners in outdated categories?

If we choose bridges, then we must be careful not to dig trenches between health and illness – even in forensic settings – for we may end up falling into them ourselves. In choosing bridges over trenches, psychiatry can reclaim its potential as a healing discipline that honors complexity rather than simplifying it to exclusion.
